# A Team-Based Training for Continuous Glucose Monitoring in Diabetes Care: Mixed Methods Pilot Implementation Study in Primary Care Practices

**DOI:** 10.2196/45189

**Published:** 2023-04-24

**Authors:** Melissa K Filippi, Angela M Lanigan, Sean M Oser, Jillian Alai, Alicia Brooks-Greisen, Tamara K Oser

**Affiliations:** 1 American Academy of Family Physicians Leawood, KS United States; 2 Department of Family Medicine University of Colorado School of Medicine Aurora, CO United States

**Keywords:** continuous glucose monitoring, CGM, continuing medical education, CME, diabetes, evaluation, family physician, implementation, primary care, primary care practice, glucose, glucose monitoring, pilot study, workflow, medical education

## Abstract

**Background:**

The American Academy of Family Physicians (AAFP) develops and maintains continuing medical education that is relevant to modern primary care practices. One continuing medical education modality is AAFP TIPS, which are comprised of resources designed for family medicine physicians and their care teams that aid in quick and accessible practice improvement strategies, with actionable steps. Evaluating physicians’ use of and satisfaction with this modality’s content and implementation strategies has not been prioritized previously. Continuous glucose monitoring (CGM) plays an increasing role in the treatment of diabetes; uptake occurs more rapidly in endocrinology settings than in primary care settings. To help address such differences in CGM uptake and diabetes care, AAFP TIPS on Continuous Glucose Monitoring (AAFP TIPS CGM) was developed, using published evidence and input from content experts (family medicine faculty; AAFP staff; and an advisory group comprised of other primary care physicians, patients, a pharmacist, and a primary care practice facilitator). A pilot implementation project was conducted in 3 primary care practices.

**Objective:**

To evaluate AAFP TIPS CGM in primary care practices, the research team assessed use of and satisfaction with the content and assessed barriers to and facilitators for strategy and workflow implementation.

**Methods:**

In total, 3 primary care practices participated in a mixed methods pilot implementation of AAFP TIPS CGM between June and October 2021. Practice champions at each site completed AAFP TIPS CGM and baseline practice surveys to evaluate practice characteristics and CGM prescribing. They conducted team trainings (via webinars or in person), with the goals of implementing CGM into practice and establishing or improving CGM workflows. Practice champions and team training participants completed posttraining surveys to evaluate the training, AAFP TIPS materials, and likelihood of implementing CGM. Interviews were conducted with 6 physicians, including practice champions, 2 months after team training. Satisfaction surveys were also distributed to those who completed the AAFP TIPS CGM course via the internet during the study period.

**Results:**

Of the 3 practices, 2 conducted team trainings. The team training evaluation survey showed that practice staff understood their role in implementing CGM in practice (19/20, 95%), and most (11/20, 55%) did not have questions after the training. Insurance coverage for CGM was a remaining knowledge gap and potential barrier to implementing CGM in practice. Physicians and interdisciplinary care team members who took the AAFP TIPS CGM course via the internet, as well as those who attended in-person team training, expressed a high degree of satisfaction with the education, content, and applicability of the course.

**Conclusions:**

This pilot implementation of AAFP TIPS CGM offers pertinent and timely information for primary care practices that desire to initiate or expand CGM use to best meet the needs of their patients with diabetes.

## Introduction

Continuous glucose monitoring (CGM) is recommended for many people with diabetes [1,2], including those with type 1 diabetes, type 2 diabetes treated with insulin, and type 2 diabetes treated with noninsulin regimens. CGM use continues to expand in different populations of people with diabetes [3-11]. Most people with diabetes receive their diabetes care in primary care settings [12,13]. Therefore, it is important to provide primary care physicians and advanced clinicians with education and tools for implementing CGM.

CGM is associated with improved hemoglobin A_1c_ levels, decreased hypoglycemia, and improved quality of life, and it can reduce or replace the use of finger sticks for the self-monitoring of blood glucose [14,15]. The use of CGM can facilitate shared decision-making and treatment decisions between primary care teams and patients with diabetes [16]. CGM uptake in endocrinology and diabetes subspecialty settings has been higher than that in primary care settings [17-20]. Since most people receive their diabetes care in primary care settings [12,13], there have been recent efforts and resources for facilitating CGM implementation in primary care settings. The American Academy of Family Physicians (AAFP) TIPS on Continuous Glucose Monitoring (AAFP TIPS CGM) is one such effort.

AAFP TIPS are brief, interactive, web-based continuing medical education (CME) courses that focus on team-based tools and customizable team training slide decks, which are designed to assist physicians and care teams in making immediate practice improvements [21]. Team-based learning is a preferred method of learning for primary care and other specialties because care delivery requires effort from multiple stakeholders and not just from individual physicians [22-28]. AAFP TIPS CGM was created to assist primary care physicians and clinicians in implementing CGM into practice workflows (ie, processes required for CGM prescribing and care). This paper describes barriers to and facilitators for a CGM pilot implementation in primary care practice workflows, including the use of and satisfaction with AAFP TIPS CGM content, and lessons learned for designing future CGM content that is tailored to primary care. This study is imperative, as it is the first implementation evaluation performed on the AAFP TIPS team-based learning platform.

## Methods

### Intervention

**Figure 1:**
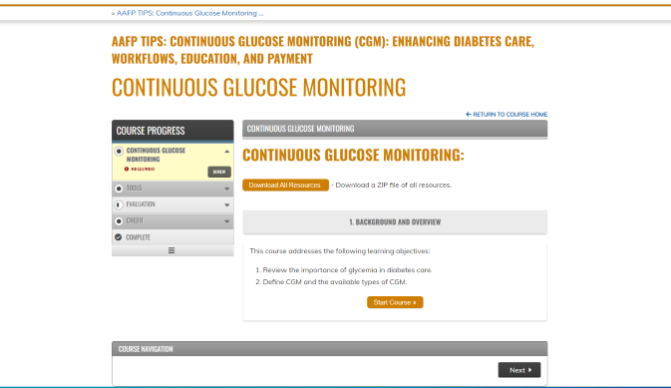
Screenshot of the AAFP TIPS CGM course landing page. AAFP TIPS CGM: American Academy of Family Physicians TIPS on Continuous Glucose Monitoring.

This mixed methods pilot implementation of the web-based AAFP TIPS CGM course assessed primary care practice adoption, experience, and feedback during the immediate 6 months following the course’s launch (April 16, 2021, through October 19, 2021). The course has a variety of education topics and resources (Table 1 and Figure 1).

A physician practice champion enrolled in and completed AAFP TIPS CGM. Team-based trainings, which roughly mirrored course lessons, were designed to be customizable by practice champions. Each slide deck took about 30 minutes to present. Practice champions were encouraged to present a team training within 30 days of completing the course, utilizing the slide decks and facilitator guide. They ultimately determined how and when to implement team trainings and which resources and slide decks to present.

**Table 1:** AAFP TIPS CGM^a^ lesson topics and tools.

Lesson	Topics	Tools (unless noted, tools are practice facing)
Lesson 1	Background and overview of CGM^b^ Types of CGM	CGM overview handout
Lesson 2	Overview of American Diabetes Association Standards of Medical Care in Diabetes Case studies Identifying patients who may benefit from CGM	Patient CGM identification tool (handout) Perceived benefits and burdens of CGM scales (surveys)
Lesson 3	Educating patients about CGM Shared decision-making Interpreting CGM data and reports Using CGM to inform treatment adjustments Case examples	Components of a continuous glucose monitor (handout) Patient-facing DiabetesWise.org website guide (handout) Key CGM measures (handout) Patient-facing handout
Lesson 4	Practice workflow and integration Ordering CGM Documentation Billing Follow-up Keeping updated Quality improvement	Sample prescription order for CGM supplies Sample CGM practice checklist for personnel (workflow) Coding and billing for CGM (handout) *The Case for CGM* (PowerPoint [Microsoft Corporation] team training presentation) *Understanding CGM* (PowerPoint team training presentation) CGM Staff Roles (PowerPoint team training presentation) Facilitator guide for roles, tasks, and workflow activities *CGM Quality Improvement* (PowerPoint team training presentation) Quality improvement planning worksheet CGM run chart Helpful links Patient registry example

^a^AAFP TIPS CGM: American Academy of Family Physicians TIPS on Continuous Glucose Monitoring.

^b^CGM: continuous glucose monitoring.

### Participants and Setting

A total of 3 primary care practices from the AAFP National Research Network were selected based on their interest and their ability to implement training within the study period. Physician champions at each site were both participants of this study and part of the team executing the intervention because of their role in teaching the team training. Interviews were conducted with physician champions and additional physicians at one site.

Beyond the three participating practices, the course was available to the public from April 16, 2021, until April 15, 2023.

### Outcome Measures

Practice champions completed a baseline practice survey about practice characteristics, the patient population with diabetes, and CGM use. Upon completion of a team training, practice champions and team training participants completed an anonymous, web-based evaluation that assessed satisfaction with course materials and comfort with implementing CGM workflows. Practice champions completed a CGM use postsurvey 2 months after team training. Physician training participants were invited to participate in a semistructured interview.

Course satisfaction was measured with a standard CME evaluation survey that is included in AAFP CME. Everyone who completed the final lesson of the course was presented with an optional CME evaluation. Evaluations completed between April 16, 2021, and September 30, 2021, were included in the analysis.

### Analysis

The data analysis included descriptive statistics for surveys and a deductive analytical approach for interview text (ie, rapid qualitative analysis) [29,30].

### Ethics Approval

This study was approved by the AAFP Institutional Review Board (protocol 21-412). Physician champions were consented via electronic consent. A waiver of documentation of informed consent was granted for survey respondents and interviewees. Survey respondents received a US $25 e–gift card, and interviewees received a US $100 e–gift card. All data were deidentified and stored on a secure server.

## Results

### Survey Results

Participating sites represented diverse practice types (a large multispecialty practice, a family medicine residency practice, and a single-physician practice). Table 2 presents practice characteristics, which were measured with survey responses.

Each site conducted customized team trainings that varied in terms of length, content, and attendees. The solo practice did not conduct formal team training, given the team’s small size. Physicians and interdisciplinary care team members who attended a team training reported a high degree of satisfaction with the training, content, and applicability of information (Table 3). Practice champions did not report how many people attended training at each site or how many declined to attend. We also do not know if other physicians or staff at each site completed the course via the internet. Practice staff who completed the team training evaluation (n=20) reported understanding their role in a CGM workflow (19/20, 95%). Over half of the respondents (11/20, 55%) indicated that they had no questions or concerns about CGM or CGM workflows, and 25% (5/20) reported questions or concerns about insurance coverage (Table 4). In accordance with AAFP standards for web-based CME evaluation, no posttraining knowledge test was given.

**Table 2:** Practice demographics of evaluation sites (n=3).

Site number	Practice type	Clinicians at the site, n	Patients by sex in clinics, %		Greater than 20% of practice population includes racial minority patients	Greater than 10% of patient population includes Hispanic or Latinx patients	Payer mix
			Male	Female			
1	Primary care only	1	40	60	Yes	Yes	Medicare: 40%; Medicaid: 15%; both Medicare and Medicaid: 0%; commercial insurance: 40%; uninsured: 5%; other: 0%
2	Community-based residency center	≥21	51	49	Yes	No	Medicare: 20%; Medicaid: 60%; both Medicare and Medicaid: 10%; commercial insurance: 7%; uninsured: 3%; other: 9%
3	Academic-based residency center	≥21	46	54	Unsure	Yes	Medicare: 19%; Medicaid: 5%; both Medicare and Medicaid: 5%; commercial insurance: 70%; uninsured: 0%; other: 1%

**Table 3:** Team training satisfaction results (n=20).

Items	Ratings					
	Rating, mean (SD)	5, n (%)	4, n (%)	3, n (%)	2, n (%)	1, n (%)
How do you rate the training materials (PowerPoints, handouts, and interactive activities) overall?^a^	1.45 (0.6)	0 (0)	0 (0)	1 (5)	7 (35)	12 (60)
The training met my expectations^b^	1.2 (0.41)	0 (0)	0 (0)	0 (0)	4 (20)	16 (80)
I will be able to apply the knowledge I learned^b^	1.2 (0.41)	0 (0)	0 (0)	0 (0)	4 (20)	16 (80)
The content was organized and easy to follow^b^	1.35 (0.67)	0 (0)	0 (0)	2 (10)	3 (15)	15 (75)
The materials distributed were pertinent and useful^b^	1.2 (0.41)	0 (0)	0 (0)	0 (0)	4 (20)	16 (80)
Class participation and interaction were encouraged^b^	1.15 (0.37)	0 (0)	0 (0)	0 (0)	3 (15)	17 (85)
Adequate time was provided for questions and discussion^b^	1.35 (0.67)	0 (0)	0 (0)	2 (10)	3 (15)	15 (75)
I understand what role I will play in CGM^c^ workflows^b^	1.4 (0.6)	0 (0)	0 (0)	1 (5)	6 (30)	13 (65)

^a^Ratings: 5=very poor; 4=poor; 3=average; 2=good; 1=excellent.

^b^Ratings: 5=strongly disagree; 4=disagree; 3=neutral; 2=agree; 1=strongly agree.

^c^CGM: continuous glucose monitoring.

**Table 4:** Team training results (n=20).

Question and responses		Respondents, n (%)
**After taking this training or series of trainings about CGM^a^, what questions and/or concerns do you have? (Mark all that apply)**		
	I have no questions or concerns	11 (55)
	I have questions or concerns about how CGM works	3 (15)
	I have questions or concerns about how to introduce CGM to patients	2 (10)
	I have questions or concerns about how to interpret CGM data	2 (10)
	I have questions or concerns about my role/responsibilities in the CGM workflow	2 (10)
	I have questions or concerns about insurance coverage for CGM for our practice's patients	5 (25)
	I have questions or concerns about billing or coding for CGM	2 (10)
	I have other questions or concerns (please specify)	0 (0)

^a^CGM: continuous glucose monitoring.

During the study period, 376 individuals completed AAFP TIPS CGM. Respondents who completed the CME evaluation (n=117) expressed high levels of satisfaction with the education, tools, and resources. An overwhelming majority (112/116, 96.6%) agreed or strongly agreed that they would recommend the activity to colleagues (Table 5). Respondents to this survey were not necessarily study participants, and we were unable to determine if overlap existed between respondents and our team training evaluation.

**Table 5:** AAFP TIPS CGM^a^ CME^b^ evaluation survey results (n=117).

Items	Poor or strongly disagree, n (%)	Fair or disagree, n (%)	Good or neutral, n (%)	Very good or agree, n (%)	Excellent or strongly agree, n (%)
How well did this activity provide you with practical knowledge or strategies you can immediately apply to your practice?^c^	0 (0)	2 (1.7)	10 (8.5)	45 (38.5)	60 (51.3)
How would you rate the effectiveness of the tools?^c^	0 (0)	1 (0.9)	12 (10.4)	37 (32.2)	65 (56.5)
How well did this activity address barriers to your optimal patient management?^c^	0 (0)	3 (2.6)	19 (16.5)	42 (36.5)	51 (44.3)
I would recommend this activity to my colleagues^d^	0 (0)	1 (0.9)	3 (2.6)	45 (38.8)	67 (57.8)

^a^AAFP TIPS CGM: American Academy of Family Physicians TIPS on Continuous Glucose Monitoring.

^b^CME: continuing medical education.

^c^The responses for this item were “poor,” “fair,” “good,” “very good,” and “excellent.”

^d^The responses for this item were “strongly disagree,” “disagree,” “neutral,” “agree,” and “strongly agree.”

### Interview Results

A total of 3 practice champions and 3 other physicians at the community residency site were interviewed. Due to scheduling challenges, we were not able to interview physicians or clinicians at the academic residency. Physician interviewees (n=6) had little to no previous experience with prescribing CGM, but some had seen patients using a continuous glucose monitor prescribed by a specialist. Overall, they expressed the desire to be better informed about how to successfully prescribe CGM, answer patients’ questions, and interpret CGM data to potentially improve patient outcomes.

Interviewees reported perceived benefits of CGM prescribing and CGM data interpretation, including alleviating reliance on specialists, bolstering decision-making with patients, influencing patient outcomes and behavior change, aiding adherence, and preventing discomfort issues with traditional blood glucose monitoring finger sticks.

With regard to course utility, participants thought that the course provided foundational knowledge for clinical care and data interpretation. Interviewees cited the *Interpreting CGM Data and Reports*, *Identifying Patients Who May Benefit from CGM*, and *Case Studies* lessons as the most useful lessons. The tools and resources were reported as helpful, but not all interviewees used every tool.

CGM prescribing workflows varied across sites because of differences in practice models, staffing, patient needs, and impacts of the COVID-19 pandemic. Interviewees cited barriers (eg, prior authorizations and insurance coverage, issues with workflow refinement and follow-up, and patient hesitancy) for incomplete CGM implementation at the time of interviews.

### Suggestions

Interviewees provided suggestions for course improvement, including expanding case studies (eg, electronic health record prescription navigation, patients with cognitive limitations or cognitive decline, and patients with financial concerns), adding more brand-specific information, including more professional CGM information, and refining some tools to specify physician tasks and staff tasks. Interviewees also suggested more information on insurance authorization processes and local and regional particularities that impact obtaining continuous glucose monitors. Lastly, they wanted manufacturer resources (eg, samples and videos).

## Discussion

### Principal Findings

This study describes a pilot implementation of AAFP TIPS CGM—a course designed for physicians and care teams to initiate or expand CGM use in primary care practices. Respondents reported being highly satisfied with and informed by the course materials. Sites were able to modify or create new CGM prescribing workflows. Interviewees also had suggestions for additional content (eg, information on successful prescription and insurance authorization [31-33], strategies for helping uninsured or underinsured patients or those with limited means [33], greater detail on professional CGM [20,34-36], electronic health record navigation [33], and the use of samples [37]).

### Limitations

This study has limitations. This study’s short duration, which was compounded with the implementation of new workflows during the COVID-19 pandemic, contributed to difficulties in implementing new steps. Further, navigating insurance authorizations presented challenges, and no one utilized billing and coding tools or practice checklist tools. Additionally, team trainings were inconsistent across sites. Practice champions developed in-person trainings, and sites did not include all training sections. We also did not measure pre- and post-CGM knowledge. Lastly, our sample of practices leaned heavily on residency programs.

### Conclusions

This pilot implementation offers pertinent and timely information for primary care practices seeking to initiate or expand CGM use to best meet the needs of their patients with diabetes. Although course information was relevant and useful, additional resources and strategies, especially those for insurance coverage and authorizations, would be helpful for successful CGM workflow implementation.

## Abbreviations

AAFP: American Academy of Family Physicians
AAFP TIPS CGM: American Academy of Family Physicians TIPS on Continuous Glucose Monitoring
CGM: continuous glucose monitoring
CME: continuing medical education

## Data Availability

The data are hosted on the research team’s internal servers and can be provided under reasonable request.
